# Psychological outcomes from a citizen science study on microplastics from household clothes washing†

**DOI:** 10.1039/d5va00037h

**Published:** 2025-05-13

**Authors:** Cameron Brick, Anna Bosshard, Bernou Boven, Julia Hijink, Antonia Praetorius, Lies Jacobs

**Affiliations:** a Department of Psychology, University of Amsterdam Amsterdam The Netherlands c.brick@uva.nl; b Department of Psychology, University of Inland Norway Elverum Norway; c Department of Ecosystem and Landscape Dynamics, Institute for Biodiversity and Ecosystem Dynamics, University of Amsterdam Amsterdam The Netherlands; d Department of Earth and Environmental Sciences, KU Leuven Leuven Belgium

## Abstract

Microplastic pollution in the form of synthetic microfibers is an increasing concern to human and ecological health, and household clothes washing is a major contributor to microplastic emissions. Consumer choices and washing behaviors could reduce this pollution, yet the psychological and behavioral drivers of these actions remain unknown. We present a pre-registered, three-month citizen science project in which Dutch residents used microfiber-capturing laundry bags at home. The citizen scientists completed pre- and post-study surveys of psychological factors such as identity, norms, perceived responsibility, and intentions, as well as washing behaviors like load size and washing temperature. After the study, citizen scientists increased modestly in problem awareness and perceived responsibility, but there were no significant changes in identity, personal norms, social norms about sustainability, perceived behavioral control, or intentions to use a laundry bag. To assess generalizability, we also compared the citizen scientists to a control sample of urban Dutch residents. The washing behaviors were weakly or uncorrelated with demographics or with psychological factors, suggesting that interventions on washing behaviors might focus on habits and skill development rather than trying to increase pro-environmental motivation. These results also suggest that interventions tested in citizen scientists may translate better to other populations than was previously suggested. Citizen science is a viable method for studying household washing under real-world conditions and provides insights for designing targeted behavioral interventions.

## Abstract 2

Washing clothes releases microfibers, but people lack tools to reduce this pollution. This field study uses citizen science to identify behavior change targets and test learning through participation.

## Graphical abstract

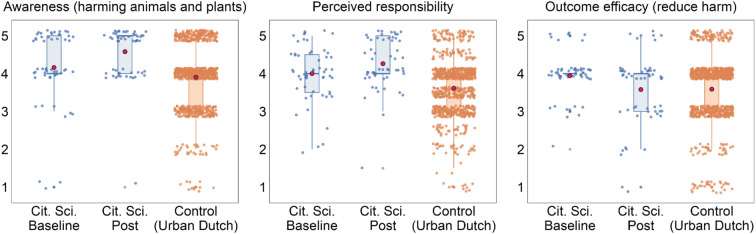

**Environmental significance**

Microplastic pollution is an accelerating environmental threat, with synthetic microfibers from household laundry contributing significantly to aquatic and terrestrial contamination. These persistent pollutants enter ecosystems, are ingested by organisms, and have toxicological effects. While technological solutions exist, their effectiveness depends on widespread behavioral adoption, yet little is known about the psychological and behavioral factors that drive such changes. This study shows how citizen science can be used as a tool for both data collection and behavior change and to assess whether participation shifts environmental attitudes and actions. The findings here challenge assumptions that citizen science automatically fosters pro-environmental behavior, highlighting the need for targeted, evidence-based interventions to reduce microfiber emissions at the household level.

Microplastics are tiny pieces of plastic (≤5 mm) in the air, land, or water that mostly result from the breakdown of consumer products and industrial waste. Microplastic pollution is accelerating and uniquely threatens ecosystems due to its persistence, potential for ingestion by organisms, and adverse effects.^1^ These fragments also threaten human health after being inhaled or ingested.^2^ A major source of household emissions of microplastics is textiles,^3^ particularly the synthetic microfibers generated during textile washing.^4,5^ One class of technological solutions to reduce textile-related microplastic emissions includes promoting natural fibers (cotton, wool, silk) in textile manufacturing, as well as ‘semi-synthetic’ fibers made from bio-based feedstocks (*e.g.*, wood pulp).^6^ However, natural fibers often contain chemical additives, and semi-synthetic fibers have been chemically transformed such that they often resemble synthetic fibers, *e.g.*, they have low biodegradability. Regardless, the vast majority of textiles are still made from fossil fuels (*e.g.*, polyester, acrylic, and nylon). So long as a majority of textiles are made from traditional plastics, consumer behavior represents a meaningful opportunity to limit microplastic emissions from households. Increasing consumer awareness of microplastic sources by actively involving participants in research on microfiber release during home washing could yield multiple benefits, including a reduction in microplastic emissions.

## Citizen science

We define *Citizen science* as public participation in scientific research and knowledge production,^7^ similar to community-based monitoring and participatory research. Citizen science has a high potential for addressing sustainability challenges through the combination of democratization and productivity goals.^8^ Democratization relates to the potential capacity of citizen science to help share decision making power and facilitate a two-way dialogue between scientists and citizens during the research process.^9^ Productivity refers to the increased potential to study environmental issues because citizen science can provide more data across diverse locations and across time. For example, most environmental Sustainable Development Goal (SDG) indicators lack data, and citizen science is already contributing to SDG monitoring.^10^ The study of household microplastic emissions particularly informs SDG 6: clean water and sanitation. Overall, citizen science appears to be a reliable, affordable, and scalable tool for research projects on pollution sources and levels.^8^

Citizen science is often claimed to facilitate transformative change around environmental issues, such as in a white paper by the European Union,^11^ but it is not clear whether this change is occurring. This is important because the answer informs whether to prioritize study designs including citizen science. There are two key inferential gaps: (1) citizen science programs may have transformative potential particularly for individual skills, but transformation may not occur at the organizational and institutional levels; and (2) there are wide claims that participation increases learning, but the claims about transformation are more often based on assumptions than evidence because learning often goes unreported or unevaluated.^12^ These issues persist because impact assessment on participants is still rare: a review of 77 citizen science projects reported that only 16% reported baseline, outcome, or impact data.^13^ Another review of 31 citizen science projects on environmental topics found that participation primarily enhanced skills and knowledge more than changing attitudes, values, or behaviors, and that only 26% of projects measured behavior.^14^ Overall, it was rare that citizen science projects measured attitudes, knowledge, and/or behavior before and after participation.^15,16^ In sum, citizen science is providing a new interface between scientists and the general public, but it remains unknown whether this contact changes the participants, or what the consequences are for individual behaviors that affect the environment.

From a measurement validity perspective,^17^ most citizen science impact assessments were ad-hoc, rarely involved psychologists or experts on survey design, and therefore had substantial measurement problems. For example, in one review the psychological predictors like values correlated above *r* = 0.4 with environmentally significant behaviors, which represents a very large relationship with a behavior.^14^ In environmental psychology, such magnitudes are usually seen when the behavior measures are vague, Likert-type self-reports that were assessed similarly to the attitudes or values measures.^18,19^ We would not expect such large correlations beyond shared measured variance because pro-environmental behaviors such as clothes washing choices are driven also by physical context and other people.^20^ Helpfully, validity concerns are being raised in citizen science impact assessment,^21^ and more attention to validity would strengthen the quality of future studies. Among related fields, environmental psychology may be uniquely poised to contribute expertise in psychometric validation, survey design, and behavior measurement to these citizen science efforts.

## Impact assessment goals

In environmental psychology, there is no dominant overarching theory. A recent commentary^22^ encouraged a shift from traditional deductive approaches toward more descriptive and inductive research. We designed this study and the research goals with these inductive goals in mind, *e.g.*, in prioritizing a multiple time point design with hands-on behaviors (not just self-report).

There are three psychological inferences it would be valuable to test in citizen science projects on environmental issues. First, the effectiveness of citizen science projects aligns with core theories on pro-environmental behavior like the norm activation model^23^ and value-belief-norm theory.^24^ Intensive hands-on engagement with environmental issues could make people more aware, foster a sense of responsibility, and sense that their actions can make a difference. Strong tests require causal inference and would be greatly aided by multiple time periods of data (*i.e.*, pre- and post-tests). Second, the relevance of different person characteristics for motivating action can vary across behaviors and context.^20^ Thus, testing is needed to establish whether to focus impact assessments or interventions on environmentalism (*e.g.*, pro-environmental norms and identities), perceptions about a problem (*e.g.*, awareness and responsibility for microfiber pollution), or beliefs about the behavior (*e.g.*, whether the behavior is perceived as effective, easy, or having negative side effects). The third key inference we suggest testing is whether citizen scientist results are generalizable. The citizen science literature is rapidly expanding, but demographic and psychological characteristics are rarely compared to outside samples *cf.*^25^ Limited evidence suggests that citizen scientists are more environmentally concerned, more White, and disproportionately come from more economically advantaged areas.^26,27^ Such comparisons are necessary to infer that the results also apply to different communities and could be used to design effective interventions and policies for the general public.

## Current study

We present one of the first studies on microfiber pollution from home washing in partnership with citizen scientists.^28^ Until now, it has been unclear what types of individuals in what situations are willing to engage in pro-environmental washing behaviors such as effortfully filtering microplastics. To address this challenge, we present a pre-registered study.^29^ We recruited citizen scientists in the Amsterdam region of Netherlands to conduct at-home capturing of microfiber emissions from clothes washing using laundry bags. They not only collected data, but engaged in self-education, behavioral skills training, and impact assessment through pre- and post-surveys. Additionally, we surveyed a large comparison group from urban regions of the same country to compare the demographics and psychological characteristics with the citizen scientists, and also to investigate with more statistical power which psychological factors were associated with intentions and behavior.

### Hypothesis 1

After completing the citizen scientist project and monitoring their microplastics, citizen scientists will report more environmentalist identity, social norms about sustainability, knowledge of microfiber pollution, awareness about the harms of microfiber pollution, perceived responsibility, outcome efficacy, personal norms, perceived behavioral control, and intentions to use laundry bags.

### Hypothesis 2

The citizen scientists compared to the sample of urban residents of the Netherlands will be more female and educated (2a), and have higher environmentalist identity, social norms about sustainability, objective knowledge of microfiber pollution, awareness of harms, perception of responsibility, outcome efficacy, personal norms, perceived behavioral control, and positive attitudes towards laundry bags (2b).

### Hypothesis 3

The intention to use laundry bags, watching a video on microfiber pollution for more time, reporting lower washing temperatures, wearing clothing more before washing, more time between washing, and washing more full loads will be positively associated with being younger, female, and more educated (3a) and the psychological factors in Hypothesis 1 (3b), tested in a separate online sample. After the pre-registration, the order and numbering of the hypotheses was updated for clarity, but the content of the hypotheses stayed the same.

## Method

### Design and procedure

The analysis plan was pre-registered at https://osf.io/edwum and all deviations are disclosed. All participants gave informed consent, and the study received ethical approval from the University of Amsterdam (2022-SP-15548, 2022-SP-15651).

### Citizen scientists procedure

**Fig. 1:**
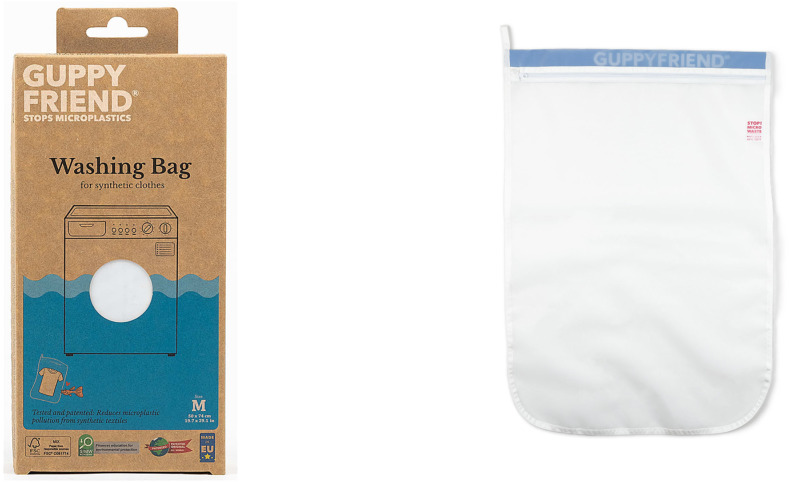
The Guppy Friend® Laundry Bag.

This study included a 3-month intervention and an online survey. After a baseline survey, during a period of around three months participants were asked to wash their clothes 10 times in a Guppy Friend® laundry bag (Fig. 1). These bags collect microfibers normally released into wastewater during home washing. After each washing cycle, participants collected the emitted microfibers from the bag with a lint roller and sent a picture of the lint sheet to the researchers for fiber analysis. For each washing cycle, participants also reported their washing machine settings and the type, weight, and textiles of the washed garments *via* an online form. Finally, participants repeated the baseline survey.

### Control survey procedure (Urban Dutch)

The control participant only responded to a cross-sectional online survey without performing the physical data collection with washing bags. The purpose of this group was to assess the generalizability of the citizen scientist results and to provide well-powered tests of the associations between psychological predictors and pro-environmental washing intentions and behaviors.

## Participants

### Citizen scientist participants

**Fig. 2:**
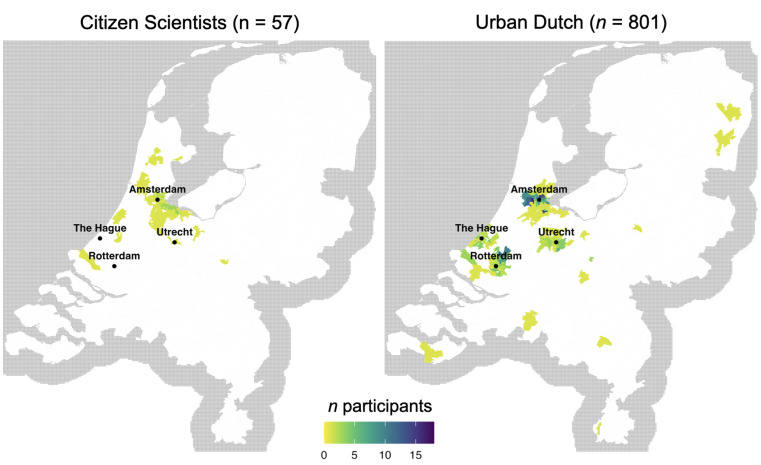
Participant residence from Dutch postcodes. Note. 13 participants of the control survey (Urban Dutch) (*n* = 814) did not provide postcodes.

One hundred participants, at least 16 years old and who lived in the Netherlands, were recruited through the researchers' networks, emails to environmental organizations, Twitter/X posts, printed flyers at the university, and the psychology student participant pool. In sum, this convenience sample was recruited with digital and physical advertisements (see OSF for these files). Most participants lived in Amsterdam or the surrounding area (Fig. 2); that the participants had a relationship with the Amsterdam metropolitan region was preferred by the funder. Psychology students could earn six research credits. The final sample of 57 participants completed the three-month (10 washes) citizen science project and the follow-up survey (43 more only completed 1–2 washes and/or did not complete the final questionnaire and were excluded). The mean age of the final sample of 57 was 39.5 years (SD = 17.5). Of the participants, 41 identified as women (72%), 12 as men (21%), two as non-binary or gender fluid (4%), and the gender identity of two was not provided (4%).

#### Power analysis (Hypothesis 1)

The desired sample size was determined based on recruitment feasibility (*N =* 100). An *a priori* power analysis in the R package *pwr* was run with alpha = 0.05 and *N* = 100. Based on the final sample of *N* = 57 after attrition, a second power analysis with G*Power^30^ suggested 80% power to detect pre-post effects of |*d*| ≥ 0.33 and 95% power to detect effects of |*d*| ≥ 0.44 (one-sided, Wilcoxon signed-rank test, matched pairs).

#### Power analysis (Hypothesis 2)

A sensitivity power analysis (not pre-registered due to an oversight) suggested 80% power to detect differences of |*d*| ≥ 0.34 and 95% power to detect differences of |*d*| ≥ 0.45 between the citizen scientists (*n* = 57) and control participants (*n* = 814)

### Control survey participants (Urban Dutch)

The control group of urban Dutch residents completed a cross-sectional survey on washing habits and environmental attitudes, which helps assess the generalizability of the citizen scientist data. Participants who lived in the Netherlands and were at least 16 years old were recruited through the online platform Panelclix and were compensated 1 EUR. To enhance comparability with the citizen scientists, we targeted residents of large Dutch cities (Amsterdam, Rotterdam, The Hague, Utrecht). Fig. 2 shows participant residence based on postcodes. The analyzed sample included 814 control participants, *M* (SD) age = 47.4 (16.1) years. 428 participants (53%) identified as women, 284 (35%) identified as men, and two (<1%) identified as non-binary. The gender identity of the remaining participants was either unclear (28 participants, 3%) from the open text responses or not provided (72 participants, 9%).

#### Power analysis (Hypothesis 3)

The sample size was determined by available funding and by the sample size at which correlations stabilize (*n* = 250).^31^ An *a priori* power analysis in the R package *pwr*^32^ was run for the pre-registration with alpha = 0.05 and *N* = 622. Based on the final sample of *N* = 814, a second power analysis suggested 80% power to detect correlations of |*r*| ≥ 0.10 and 95% power to detect correlations of |*r*| ≥ 0.13.

### Measures

There were two citizen scientist surveys (baseline and post-intervention), and one control survey of urban Dutch residents. Items apply to all three unless otherwise indicated and all surveys were in Dutch. For readers, we provide the originals and machine translations to English^33^ at https://osf.io/65dz4/.

### Demographics

Gender identity was measured with an open question and was coded by researchers as woman, man, non-binary or gender fluid, or missing. Age was measured using integer years. Education was measured using seven categories (highest level of completed Dutch education). Work situation was measured using 11 categories; the citizen science survey initially had 10 categories, and we added studying after coding the open responses. Location was measured with the first four numbers of Dutch postcodes, which only provides neighborhood-level resolution of a few thousand people and so preserves more privacy.

#### Washing behaviors

Participants of the control survey reported 14 features of their washing behaviors. Many laundry and clothing variables could interact in complex ways, and there is mixed evidence about their effects on microfiber emissions. We report temperature, wears tops, wears bottoms, days until full load of laundry, and typical load size here because these behaviors have the clearest environmental consequences (energy, water, and carbon emissions), and these factors are most plausibly related to microfiber emissions. The remaining impacts are shown in ESI Table S1.†

### Temperature

Participants gave percentage estimates of how often they wash at 20 °*C or lower*, 30 °C, 40 °C, 60 °C, 90 °C, and the white clothes program (hot). Lower scores are more pro-environmental.

### Days until full load

Participants indicated in integer days how long before they run a full load of laundry.

### Wears tops

Participants indicated after how many wears they wash shirts, sweaters, and blouses. The options were: after one wear,^1^ after wearing it twice,^2^ and after three or more wears.^3^

### Wears bottoms

Participants indicated after how many wears they wash pants and skirts with the same response options as for the tops.

### Washing a full load

Participants indicated how full their washing machine usually is: ¼ full (0.25), ½ full (0.5), ¾ full (0.75), or completely full.^1^

### Psychological variables

The below items were rated from strongly disagree^1^ to strongly agree^5^ unless otherwise indicated. Internal consistency of scales was assessed with McDonald's omega (*ω*). The pre-registration said that when *ω* < 0.6 for two-item scales, single face-valid items would be chosen to represent the construct, and we report robustness checks using the other items in Tables 3 and 4.

#### Environmentalist identity

We used a four-item measure.^34^ An example item was: “I identify with other environmentalists”. The scale had excellent internal consistency in the control survey (*ω* = 0.90) and good internal consistency in the citizen science surveys at baseline (*ω* = 0.86) and post-intervention (*ω* = 0.83).

#### Social norms about sustainability

We created a two-item scale based on.^35^ An example item was: “People I care about make conscious choices to reduce their environmental impacts”. The scale had good internal consistency in the control survey (*ω* = 0.78), questionable internal consistency in the citizen science survey at baseline (*ω* = 0.63), and acceptable internal consistency post-intervention (*ω* = 0.74).

#### Perceived knowledge about microfibers

We created this item ad hoc. In the control survey, the item was: “How much do you know about synthetic microfibers?”. In the citizen science baseline survey, the item was: “Before you came in contact with the […] project, which statement applied most to you?” and answers ranged from “*This is the first time I have read about synthetic microfibers*”^1^ to “*I have advanced knowledge on this topic*”.^4^

#### Objective knowledge about microfibers

We created this item ad hoc. Respondents were asked to rank four sources of microplastics according to their perceived contribution to ocean pollution. The response choices were *washing synthetic textiles*, *tyres*, *plastic packaging*, and *personal care products*, with 24 potential ranking combinations. We then scored responses on a scale from low knowledge^1^ to high knowledge,^5^ assigning ranks to predetermined combinations (see ESI†).

#### Awareness of harms of microfiber pollution

We created a two-item scale based on.^35^ An example item was: “*Microfiber release through washing harms marine animals and plants*”. The scale had acceptable internal consistency in the control survey (*ω* = 0.77), questionable internal consistency in the citizen science survey at baseline (*ω* = 0.66), and unacceptable internal consistency post-intervention (*ω* = 0.54). Following the pre-registration, only one item was used to represent the construct: “*The release of microfibers from washing harms marine animals and plants*”.

#### Perceived responsibility

We created a two-item scale based on.^35^ An example item was: “*I am partly responsible for the impact of microfibers on marine animals and plants*”. The scale had acceptable internal consistency in the control survey (*ω* = 0.79), questionable internal consistency in the citizen science survey at baseline (*ω* = 0.64), and acceptable internal consistency post-intervention (*ω* = 0.71).

#### Knowledge of laundry bags

We created these items ad hoc. In the control survey, the item was: “Have you heard of these or similar laundry bags for synthetic clothes before?”. In the baseline citizen science survey, the item was: “Except for information you might have received through the […] project, have you heard of these or similar laundry bags for synthetic clothes before?”. The response options were: “*This is the first time I have read about laundry bags for synthetic clothes*” (control survey) or “*I have read/heard about laundry bags for synthetic clothes for the first time via the […] project*” (baseline survey citizen scientists),^1^ “*I have heard about laundry bags for synthetic clothes before*”,^2^ and “*I have used laundry bags for synthetic clothes at home*”.^3^

#### Outcome efficacy

We created a two-item scale based on.^35^ The scale had unacceptable internal consistency in the control survey (*ω* = 0.03), the citizen science survey at baseline (*ω* = 0.41), and post-intervention (*ω* = 0.41). Following the pre-registration, only one item was used to represent the construct: “*Based on the information above, I think that I can reduce the harm of microfiber pollution by washing synthetic clothes in a laundry bag*”. In the post-intervention survey, the item was slightly different, starting with: “*Based on my experience over the past few weeks, I feel…*”. We provide sensitivity analyses using the alternative item (“Washing synthetic clothes in a laundry bag seems like a drop in the ocean compared to what fashion companies and retailers could do (e.g., work with less polluting materials)”.

#### Personal norm

We created a two-item scale of personal norms to use laundry bags based on.^35^ The scale had acceptable internal consistency in the control survey (*ω* = 0.72), but it was questionable in the citizen science survey post-intervention (*ω* = 0.65), and unacceptable in the baseline survey (*ω* = 0.40). Therefore, only one item was used to represent the construct: “*I feel a strong personal obligation to reduce synthetic microfiber release through washing*”.

#### Perceived effort

We planned to create a four-item scale of perceived behavioral control. The scale had poor internal consistency in the control survey (*ω* = 0.56), and it was unacceptable in the citizen science survey baseline (*ω* = 0.32) and post-intervention survey (*ω* = 0.36), and item removal did not sufficiently improve scale reliability. Therefore, only one item (“*Washing synthetic clothes in a laundry bag seems/is effortful*”) was analyzed further and for clarity we renamed the construct to *perceived effort*.

#### Attitudes beyond environmentalism

As pre-registered, we included two individual items to assess general attitudes towards laundry bags. The items were: “Washing synthetic clothes in a laundry bag seems to prevent clothes from getting cleaned properly” and “Washing synthetic clothes in a laundry bag seems to preserve clothing quality for a longer time”.

### Behaviors

#### Intention to use laundry bags

We created this item ad hoc. In the control survey, the item was: “*I intend to always use a laundry bag when washing synthetic clothes*” and in the citizen science survey, the item was: “*Beyond participating in the project, I intend to always use a laundry bag when washing synthetic clothes*”.

#### Video-watching

In the control survey, participants had the option to watch a short video about synthetic microfiber pollution and solutions.^36^ We recorded how many seconds participants stayed on the page and more time was interpreted as seeking information about environmental issues. We Winsorized the variable at 2.46 minutes (147.6 s) because that was the duration of the video (not pre-registered). Most people skipped the video (see ESI Fig. S1† for the distribution). This floor effect may have suppressed correlations with this outcome.

### Exclusions

In the control survey, participants who indicated that they were not responsible for laundry were excluded from the analyses (*N* = 62) (pre-registered). We also excluded 285 participants who completed less than half of the survey and therefore missed most key variables and excluded 18 participants who showed zero variability on the 21 items with a 5-point Likert scale (not pre-registered). These exclusions did not change the main results: see robustness tests in ESI Tables S5–S7.†

## Results

Because many variables were ordinal and some variables were non-normally distributed, we conducted non-parametric tests: Wilcoxon signed-rank tests for within-group differences (Hypothesis 1), Wilcoxon rank sum and Chi-square tests for between-group differences (Hypothesis 2), and Spearman correlations (*r*_s_) for associations (Hypothesis 3). Wilcoxon effect sizes (*r*) can be interpreted in magnitude like Pearson's correlations.^37^

Demographics are in Table 1. Consistent with Hypothesis 2a, the citizen scientists were more female (75% *vs.* 60%), *χ*^2^(1, 767) = 3.89, *p* = 0.024, and had more education, *W* = 17 844, *p* = 0.003, *r* = 0.10, than the control participants (urban Dutch). The groups had similar rates of having heard of the washing bags: citizen scientists (29%), control (19%), *χ*^*2*^(1) = 2.21, *p* = 0.14. Both groups had low rates of having used such bags: citizen scientists (4%), control (3%): no difference *χ*^*2*^(1) = 0.00, *p* = 1.

**Table 1:** Demographics

Age *M* (SD) years	Citizen scientists		Control (Urban Dutch)	
	*n* = 57		*n* = 814	
	39.5 (17.5)		47.4 (16.1)	
	*N*	%	*N*	%
**Gender**				
Woman	41	72	428	53
Man	12	21	284	35
Non-binary or gender fluid	2	4	2	<1
Unclear	2	4	100	12
Highest completed education				
Secondary school	11	19	136	17
Secondary vocational	0	0	205	25
University of applied sciences	13	23	175	21
University (Bachelor's)	8	14	90	11
University (Master's)	20	35	165	20
Doctoral degree	5	9	26	3
Other			16	2

**Occupational status (multiple possible)**				
Homemaking	12	21	321	39
Full-time employed	13	23	360	44
Part-time employed	26	46	156	19
Full-time self-employed	2	4	45	6
Part-time self-employed	8	14	43	5
Unemployed (job-seeking)	0	0	23	3
Unemployed (not job-seeking)	3	5	26	3
Retired	7	12	126	15
Unable to work	2	4	44	5
Studying	6	11	9	1
Other	1	2	4	<1

### Effects of citizen science participation

**Fig. 3:**
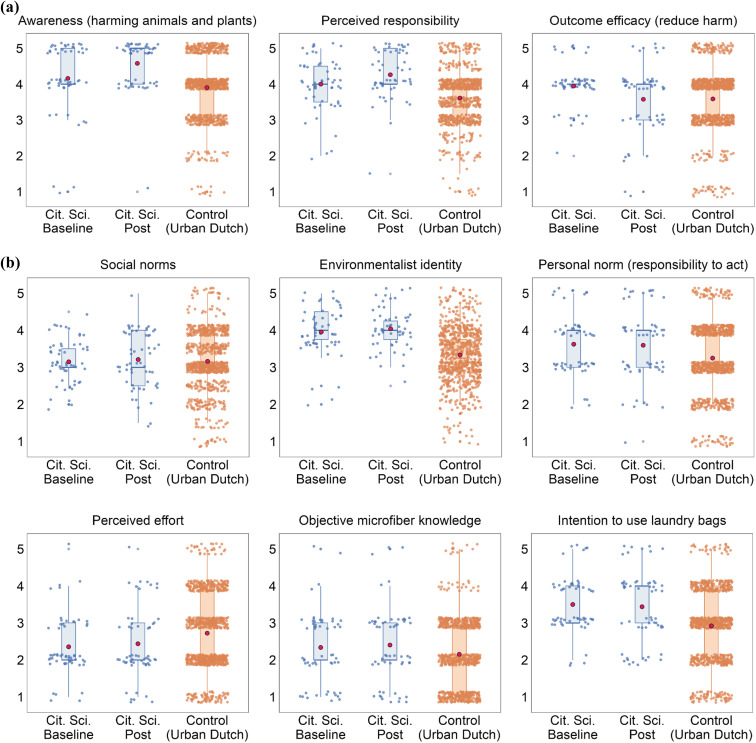
(a)Pre-registered participation outcomes with pre-post changes (*p*_s_ < 0.05). Note. The red dots indicate means, the boxes indicate interquartile ranges, and the lines in the boxes indicate medians. The points are jittered and partially transparent to better show the distributions. (b) Pre-registered participation outcomes without pre-post changes (*p*_s_ ≥ 0.05). Note. The red dots indicate means, the boxes indicate interquartile ranges, and the lines in the boxes indicate medians. The points are jittered and partially transparent to better show the distributions.

Fig. 3a and b show the key psychological variables before and after the citizen science project and Supplementary Table S2† has the descriptives. In support of Hypothesis 1, there were moderate increases in awareness about the negative impacts of microfiber emissions, *V* = 66, *r* = 0.38, *p* = 0.003, and perceived responsibility about microfiber emissions, *V* = 144, *r* = 0.35, *p* = 0.007. Against Hypothesis 1, outcome efficacy moderately decreased (*i.e.*, perceiving that the bags can make a difference), *V* = 281, *r* = 0.34, *p* = 0.005. However, this result was not robust when using another efficacy item (“*perceiving the bags as a drop in the ocean compared to actions by fashion companies and retailers*”, which we excluded from the efficacy composite due to low internal consistency; *V* = 107, *r* = 0.22, *p* = 0.07). There were no changes in environmentalist identity, *V* = 384, *r* = 0.15, *p* = 0.27; social norms about sustainability, *V* = 371, *r* = 0.10, *p* = 0.59; objective knowledge about microfiber emissions, *V* = 206, *r* = 0.06, *p* = 0.81; personal norms, *V* = 182, *r* = 0.01, *p* = 0.86; nor intentions to use laundry bags, *V* = 339, *r* = 0.06, *p* = 0.67. There were inconsistent results for the four items intended to measure perceived behavioral control, which turned out to have limited overlap. We selected ‘perceived effort’ to represent behavioral control, and it did not change, *V* = 226, *r* = 0.10, *p* = 0.67. Similarly, robustness checks of the other control items revealed no changes in the belief that bags are difficult to remember, *V* = 200, *r* = 0.18, *p* = 0.337, but after participation, citizen scientists thought the bags were somewhat more affordable, *V* = 389, *r* = 0.29, *p* = 0.046, and more durable, *V* = 96, *r* = 0.49, *p* < 0.001.

Exploratory analyses revealed no change in attitudes about the bags preserving clothing quality, *V* = 85, *r* = 0.13, *p* = 0.33, and a large decrease in concerns that the bags would prevent clothes from being properly cleaned, *V* = 335, *r* = 0.53, *p* < 0.001. ESI Fig. S3 and S4† show the robustness checks and exploratory analyses.

### Psychographic differences between citizen scientists and control participants (Urban Dutch)

Fig. 3a and b show the key psychological variables among citizen scientists and the control sample and ESI Table S3† has the descriptives. In line with Hypothesis 2b, the citizen scientists compared to control were higher in identifying with environmentalists, *W* = 11 340, *r* = 0.22, *p* < 0.001, awareness of the harms of microfiber pollution to animals and plants, *W* = 17 890, *r* = 0.10, *p* = 0.004, perceived responsibility, *W* = 13 024, *r* = 0.20, *p* < 0.001, outcome efficacy, *W* = 17 476, *r =* 0.11, *p* = 0.002, personal norms *W* = 18 255, *r* = 0.09, *p* = 008, perceived behavioral control *W* = 27 478, *r* = 0.09, *p* = 0.007, and believed less that laundry bags prevent clothes cleaning *W* = 27 562, *r* = 0.10, *p* = 0.005. However, contrary to Hypothesis 1b, there were no group differences in social norms about sustainability, *W* = 23 820, *r* = 0.01, *p* = 0.73, objective knowledge about microfiber pollution, *W* = 18 018, *r* = 0.04, *p* = 0.19, nor believing that clothes retain their quality when washed in a laundry bag, *W* = 22 644, *r* = 0.00, *p* = 0.92. ESI Table S3† has robustness checks and exploratory analyses.

### Demographic predictors of pro-environmental intentions and behaviors in the control survey (Urban Dutch)

Table 2 shows relationships between demographics, intentions to use microfiber bags, video-watching, and washing behaviors. Women reported slightly stronger intentions than men to use laundry bags, *r*_s_(710) = 0.13, *p* < 0.001, but there was no gender effect on video watching, *r*_s_(679) = −0.02, *p* = 0.63. Younger people had slightly higher intentions to use laundry bags, *r*_s_(808) = −0.08, *p* = 0.032, but older people watched moderately more of the video, *r*_s_(776) = 0.43, *p* < 0.001. Being more educated was weakly associated with more intentions to use laundry bags, *r*_s_(795) = 0.10, *p* < 0.007, but *less* video watching, *r*_s_(762) = −0.10, *p* = 0.008.

**Table 2:** Spearman correlations between demographics and intention to use laundry bags and video-watching and washing behaviors in the control survey (Hypothesis 3) (*N*_s_ = 703-814)^a^

	Range	*M* (SD)	Gender	Age	Education
			0–1	16–88	1–6
			0.60 (0.49)	47.4 (16.1)	3.03 (1.48)
Intention to use laundry bags	1–5	2.92 (0.97)	0.13	−0.08	0.10
Video-watching (sec)	0.02–2.46	0.63 (0.93)	−0.02	0.43	−0.10
Temperature (°C)	20–95	40.99 (9.39)	0.02	−0.01	−0.02
Wears tops (freq.)	1–3	2.03 (0.68)	−0.08	0.27	0.00
Wears bottoms (freq.)	1–3	2.50 (0.69)	−0.02	0.12	0.13
Washing a full load (¼ to full)	0–1	0.84 (0.18)	0.05	0.02	0.11
Days until full load	1–60	7.72 (5.78)	−0.09	0.11	0.13

a Spearman's rho correlations (*r*_s_). Correlations |*r*_s_| ≥ 0.08 are significant at *p* < 0.05, |*r*_s_| ≥ 0.10 at *p* < 0.01, and |*r*_s_| ≥ 0.12 at *p* < 0.001.

We also assessed relationships between demographics and washing behaviors: temperature, wears tops, wears bottoms, days until full load of laundry, and typical load size. Gender was unrelated to most washing behaviors, but women wore tops slightly fewer times before washing them than men, *r*_s_(711) = −0.08, *p* = 0.026, and took slightly fewer days to fill a laundry, *r*_s_(702) = −0.09, *p* = 0.024. More education was related to wearing bottoms more frequently before washing, *r*_s_(796) = 0.27, *p* < 0.001, washing fuller machines, *r*_s_(796) = 0.27, *p* = 0.001, and taking more days before doing laundry, *r*_s_(783) = 0.27, *p* < 0.001. Older people wore tops, *r*_s_(808) = 0.27, *p* < 0.001, and bottoms, *r*_s_(809) = 0.12, *p* = 0.001, more often before washing than younger people, and took more days before doing laundry, *r*_s_(794) = 0.11, *p* = 0.002. None of the demographics related to washing temperatures, all *p*_s_ > 0.59. Overall, the results did not support Hypothesis 3a.

## Psychological predictors of pro-environmental intent and washing behaviors in the control survey (Urban Dutch)

**Fig. 4:**
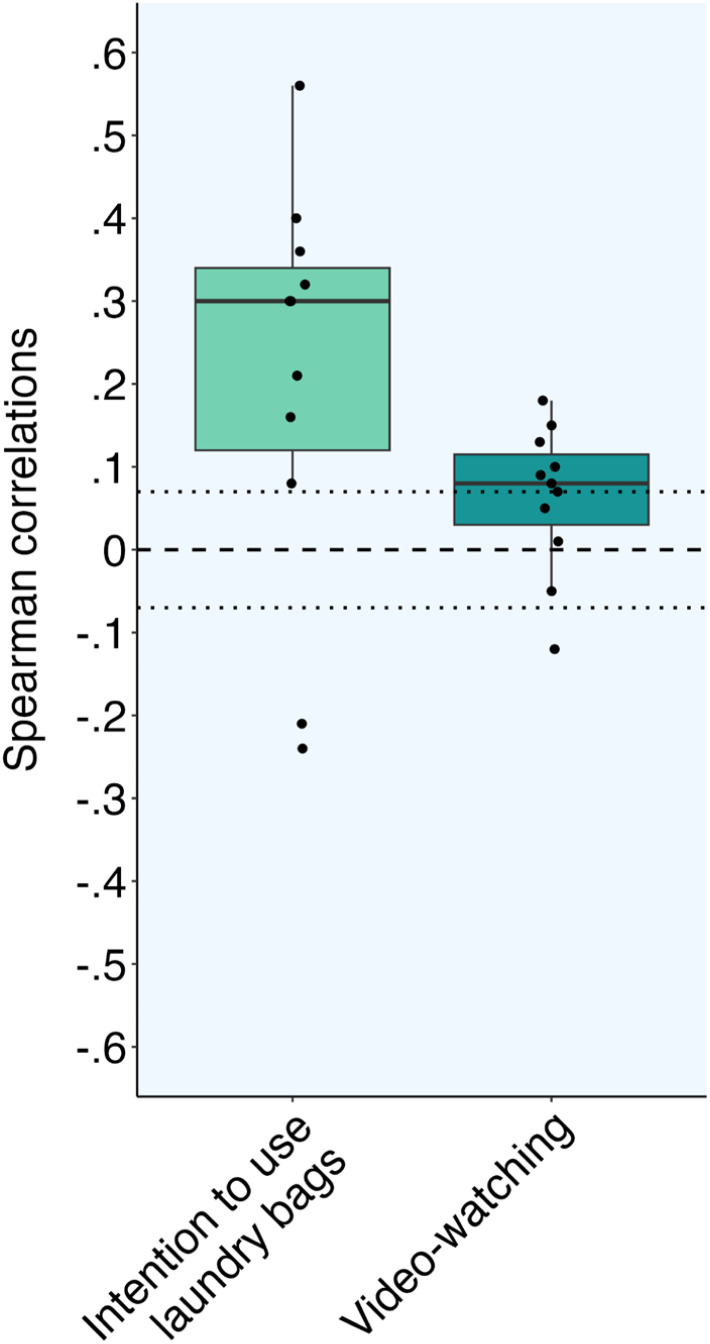
Spearman correlations between psychological variables and Intention to use laundry bags and video-watching in the control survey (Hypothesis 3) (*N*_s_ = 683-814). Note. The boxes represent the interquartile range of correlation coefficients, and each solid line represents the median correlation. The dotted lines indicate that correlations were significant at *p* < 0.05 (|*r*_s_| ≥ 0.07). 68.6% of participants stayed on the page of the video for less than 15 seconds. The distribution of video-watching is shown in Fig. S1,† and the floor effect may limit correlation size.

Table 3 and Fig. 4 show the relationships between psychological variables and intentions to use laundry bags and time spent on a video on microfiber pollution. Supporting Hypothesis 3b, all psychological variables were correlated with the intention to use a laundry bag. The strongest correlations were personal norms *r*_s_(812) = 0.56, *p* < 0.001, believing the bags are effective, *r*_s_(812) = 0.40, environmentalist identity, *r*_s_(812) = 0.36, *p* < 0.001, and believing that washing clothes in the bags preserves quality, *r*_s_(812) = 0.32, *p* < 0.001. Believing that the bags prevent cleaning, *r*_s_(812) = −0.24, *p* < 0.001, and perceived effort, *r*_s_(812) = −0.21, *p* < 0.001, were weakly negatively correlated with intentions.

**Table 3:** Spearman correlations between psychological variables and the intention to use laundry bags and video-watching in the control survey (Hypothesis 3) (*N*_s_ = 683–814)^a^

	Range	*M* (SD)	Intention to use laundry bags	Video-watching (sec)
			1–5	0.02–2.46
			2.92 (0.97)	0.63 (0.93)
Social norms	1–5	3.16 (0.79)	0.30	0.08
Environmentalist identity	1–5	3.33 (0.73)	0.36	0.18
Awareness (harming animals and plants)	1–5	3.90 (0.84)	0.21	0.15
Awareness (harming health)^robustness^	1–5	3.74 (0.84)	0.25	0.08
Perceived responsibility	1–5	3.61 (0.76)	0.30	0.10
Outcome efficacy (reduce harm)	1–5	3.59 (0.87)	0.40	0.09
Outcome efficacy (drop in the ocean)^robustness^	1–5	3.64 (0.95)	−0.14	0.01
Personal norm (responsibility to act)	1–5	3.25 (0.94)	0.56	0.13
Personal norm (right thing to do)^robustness^	1–5	3.50 (0.79)	0.54	0.08
Perceived effort	1–5	2.72 (1.02)	−0.21	−0.12
Using bags is hard to remember^robustness^	1–5	2.90 (0.98)	−0.21	−0.15
Bags are affordable^robustness^	1–5	2.53 (1.04)	0.42	0.01
Bags are durable ^robustness^	1–5	3.25 (0.70)	0.21	0.02
Bags preserve quality	1–5	3.22 (0.73)	0.32	0.05
Bags limit cleaning	1–5	3.07 (0.87)	−0.24	−0.05
Perceived microfiber knowledge	1–4	1.85 (0.75)	0.16	0.07
Objective microfiber knowledge	1–4	2.15 (0.94)	0.08	0.01

a Spearman's rho correlations (*r*_s_). Correlations |*r*_s_| ≥ 0.07 are significant at *p* < 0.05, |*r*_s_| ≥ 0.09 at *p* < 0.01, and |*r*_s_| ≥ 0.12 at *p* < 0.001.

In line with Hypothesis 3b, most psychological variables were correlated with video-watching, but these correlations were all weak. The strongest correlations were environmentalist identity, *r*_s_(779) = 0.18, *p* < 0.001, awareness of microfiber harms to animals and plants, *r*_s_(779) = 0.15, *p* < 0.001, and personal norms, *r*_s_(779) = 0.13, *p* < 0.00. Perceived effort was related to less video-watching, *r*_s_(779) = −0.12, *p* < 0.001. Last, intention to use the laundry bags was unrelated to video-watching time, *r*_s_(779) = 0.04, *p* = 0.25.

**Fig. 5:**
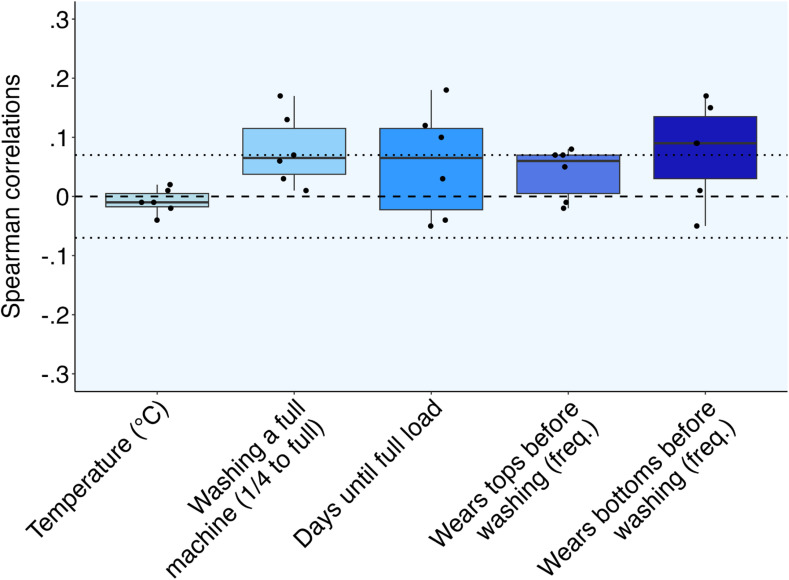
Correlations between Psychological Variables and Washing Behaviors in the Control Survey (Urban Dutch) (Ns = 702-814). Note. The boxes represent the interquartile range of correlation coefficients, and each solid line represents the median correlation. The dotted lines indicate which correlations were significant at *p* < 0.05: |*r*_s_| ≥ 0.07.

Table 4 and Fig. 5 show the relationships between psychological variables and five washing behaviors. Contrary to Hypothesis 3b, none of the psychological variables correlated with self-reported laundry temperature, all *p*_s_ > 0.24. In support of Hypothesis 3b, awareness microfiber harms to animals and plants, *r*_s_(812) = 0.07, *p* = 0.04, perceived responsibility, *r*_s_(812) = 0.08, *p* = 0.03, and perceived microfiber knowledge, *r*_s_(812) = 0.07, *p* = 0.04, were weakly related to wearing tops more frequently before washing. Moreover, environmentalist identity, *r*_s_(811) = 0.09, *p* = 0.01, awareness of microfiber harms to animals and plants, *r*_s_(811) = 0.15, *p* < 0.001, perceived responsibility *r*_s_(811) = 0.17, *p* < 0.001, and perceived microfiber knowledge, *r*_s_(811) = 0.15, *p* < 0.001 were weakly related to wearing clothes more before washing. Awareness of microfiber harms to animals and plants, *r*_s_(812) = 0.17, *p* < 0.001 and perceived responsibility *r*_s_(812) = 0.13, *p* < 0.001, were weakly related to washing clothes at full loads.

**Table 4:** Correlations between psychological variables and washing behaviors in the control survey (Urban Dutch) (*N*_s_ = 702-814)^a^

	Range	M (SD)	Temperature (°C)	Wears tops (freq.)	Wears bottoms (freq.)	Washing a full load (¼ to full)	Days until full load
			20–95	1–3	1–3	0–1	1–60
			41.0 (9.39)	2.03 (0.68)	2.50 (0.69)	0.84 (0.18)	7.72 (5.78)
Social norms	1–5	3.16 (0.79)	0.02	−0.01	0.01	0.01	−0.04
Environmentalist identity	1–5	3.33 (0.73)	−0.01	0.05	0.09	0.07	0.10
Awareness (harming animals and plants)	1–5	3.90 (0.84)	−0.01	0.07	0.17	0.17	0.18
Awareness (harming health) ^robustness^	1–5	3.74 (0.84)	−0.02	0.06	0.10	0.09	0.09
Perceived responsibility	1–5	3.61 (0.76)	−0.04	0.08	0.15	0.13	0.12
Perceived microfiber knowledge	1–4	1.85 (0.75)	0.01	0.07	0.09	0.06	0.03
Objective microfiber knowledge	1–5	2.15 (0.94)	−0.02	−0.02	−0.05	0.03	−0.05

a Spearman's rho correlations (*r*_s_). Correlations |*r*_s_| ≥ 0.07 are significant at *p* < 0.05 (apart from the correlation between environmentalist identity and washing a full load); |*r*_s_| ≥ 0.10 are significant at *p* < 0.01; and |*r*_s_| ≥ .12 are significant at *p* < 0.001.

Environmentalist identity, *r*_s_(797) = 0.10, *p* = 0.01, awareness of microfiber harms to animals and plants, *r*_s_(797) = 0.18, *p* < 0.001, and perceived responsibility, *r*_s_(797) = 0.12, *p* < 0.001, also weakly related to waiting more days until doing laundry. The other psychological variables were unrelated to these washing behaviors, all *p*_s_ > 0.06.

## Discussion

Microfiber emissions from household clothes washing is a major contributor to microplastic pollution.^3^ Changing household behavior requires engaging the public and collecting at-home data, and citizen science has a unique potential to achieve these goals.^8^ However, previous work rarely assessed whether study participation changed the individuals. We presented the first study of typical, real-world household clothes washing and microfiber filtration, and demonstrated the feasibility of citizen science for studying attitudes and behaviors over time. We also surveyed a control group of nearby residents, which allowed for testing the generalizability of the citizen scientists and higher-powered analyses of whether psychological and demographic factors were associated with pro-environmental, emissions-reducing washing behaviors. The current study was a relatively severe test of these hypotheses compared to previous work^21^ due to tools from psychology such as analysis pre-registration (less flexibility during data analysis), moderately high measurement validity during scale selection, and outcome-independent verification during analysis, which together make these results more credible.

Specifically, previous work often reported that citizen scientists learn or change from their participation, but looking closely, that learning was usually unreported or unevaluated.^12^ In contrast, the current study was longitudinal (multiple time points), and designed in collaboration with social psychologists to directly assess before-after changes in knowledge, concern, *etc.*, which responds directly to calls for greater validity in the citizen science literature.^21^ The changes we observed were relatively modest compared to previous claims, which we explain with the lack of previous validation and that many citizen scientists already reported high environmental concern, *etc.*, at baseline, leading to ceiling effects. Future work could rescale the wording of these scale items to avoid ceiling effects.

### Generalizability

The citizen scientists were more likely to be female and highly educated than the control sample of urban Dutch residents, which was consistent with Hypothesis 2a and aligns with previous findings.^26,27^ In terms of psychological factors, the citizen scientists identified more as environmentalists and perceived greater environmental responsibility than the control, and there were no significant differences in other psychological factors such as social norms about sustainability and microfiber knowledge, which provides mixed support for Hypothesis 2b. Generally, we expect that the general public would be less environmentally concerned than both of these samples that agreed to provide data for a study on microplastics and clothes washing, and neither sample was representative to the entire Dutch population, *e.g.*, the study samples had more women. Measuring these demographic and psychological differences is necessary before claiming that interventions on citizen scientists would be effective in other populations. If these demographic factors were strongly correlated with key environmental behaviors, this would be a greater concern for generalizability (see *Behavioral Insights* below).

### Impact of citizen science participation

Awareness and perceived responsibility increased modestly after citizen science participation, but there were no significant changes in psychological outcomes such as environmentalist identity, personal norms, social norms about sustainability, perceived behavioral control, or intentions to use a laundry bag to capture microfibers. We interpret the results as weak to mixed evidence for Hypothesis 2. Unexpectedly, after participation the citizen scientists perceived themselves as less capable of reducing microfiber pollution by washing clothes in a laundry bag. This finding could have been influenced by slightly different item phrasing at sign-up (“based on information [about laundry bags] you just read”) *vs.* post-intervention (“based on your experience in the past weeks”). Moreover, this finding was not robust to using an alternative item for measuring outcome efficacy (that using bags is a ‘drop in the ocean’ compared to structural changes), but it is consistent with a recent qualitative finding that when reflecting on the topic, people may feel powerless to reduce microplastic pollution.^38^ The decrease across our study may have occurred because the fiber collection process was challenging for participants: they had to use the bag, let the bag dry, use a lint roller on the inside of the bag, and then remove the lint sheet and take a picture. Because the released fibers are tiny and many are light colored against a light background, it is possible the participants did not see many fibers or much benefit from their effortful behavior. There is anecdotal support for this idea. At the conclusion of the study, we held a workshop to share and discuss the findings with the citizen scientists, and some were surprised at the amount of fibers that were visible on an enlarged, high-resolution scan of a lint sheet.

The evidence here was ambiguous about any decrease in perceived efficacy, but there are promising directions to explore any such decreases. Citizen science participants may in some cases shift their perceived responsibility for environmental issues from the individual to the collective or governmental (*e.g.*, regulation, technology, and large-scale filtration).^39^ We recommend that future work measures other types of environmental change efficacy such as governmental^40^ and see whether it also changes after citizen science participation. Overall, the citizen scientists only changed their psychological perceptions in minor ways despite the intensiveness of the intervention. The citizen scientists had high starting awareness, personal norm, and efficacy. Perhaps they believed that they could easily reduce microplastics, but participation led to being confronted with the complexity and difficulty of the problem. Further, exploratory analyses suggested that after participation, the citizen scientists saw the bags as more durable and affordable, and they were less concerned that the bags would prevent cleaning, compared to before participation. Future work could also focus on how citizen science engagement changes practical perceptions like these, rather than abstract goals like environmental protection.

### Behavioral Insights from the control group

With the large control group sample, there was enough statistical power to identify small relationships between psychological factors and intentions to use the laundry bags, watching a video about microfibers, and self-reported washing behaviors. Overall, the observed relationships with intentions were small to moderate and in the predicted direction (Table 2). The largest correlations were with intentions to use a laundry bag to capture microplastics, such as *r* = 0.5–0.6 with personal norms, outcome efficacy, and finding the bags affordable.

Participants were also given the opportunity to watch an informative video about microplastics. Most participants skipped the video (ESI Fig. S1†), and this floor effect of viewing time limited testing relationships with other factors. As expected from this restricted range, there were only modest correlations like that participants who saw themselves as environmentalists watched more of the video, *r* = 0.18. Also, participants who expected that using the bags would be effortful watched less of the video, *r* = −0.13. It is unclear from this study whether psychological factors would be important for other types of information seeking about environmental issues, but these findings do not constitute strong evidence of this claim.

Participants also reported five behaviors related to greater environmental impact of home clothes washing such as the temperature and how often they run loads. The largest relationships with a psychological predictor, and still modest in size, were that participants with more awareness of microplastic harms to animals and plants waited more days before running a full load, *r* = 0.18, and wore their tops and bottoms more before washing. Mostly, the psychological factors were unrelated to the self-reported washing behaviors. This pattern of null results is notable because of the relatively high attention in this study to validity and severe testing,^17^ and suggests that environmentalism and attitudes towards microfiber pollution may not be important for these behaviors. However, further studies with other psychological factors and improved measures of behavior (*e.g.*, beyond self-report) would be needed to make a strong claim. One possibility is that washing behavior is not perceived as particularly environmentally relevant, and therefore less moralized compared to behaviors like littering or recycling.

Across the board, control group demographics were weakly associated or not associated with the environmental behavioral outcomes. Two findings were that older participants watched the video for longer *r* = 0.43, and also reported wearing their tops (clothing) for more times prior to washing them *r* = 0.27. Overall, whether participants were female, more educated, or older did not relate much to environmentally significant decisions around clothes washing, so we reject Hypothesis 3. We infer that the demographic differences between citizen scientists and the general public in terms of gender and age, and whether this generalizability threatens inferences from the citizen scientists to the public, may therefore be only a small concern for these clothes washing behaviors. It is unknown whether such demographic differences might be more associated with other environmentally significant behaviors.

### Practical implications

This study further demonstrates that citizen science can effectively engage the public in environmental research, in this case providing valuable data on household microfiber emissions. However, the limited psychological changes observed suggest that future interventions should go beyond raising awareness, focusing instead on structural and contextual barriers, such as cost, convenience, and the perceived effectiveness of solutions to reduce microfiber emissions. For example, scientists could pivot from individual, voluntary behavior to support for policy change such as microfiber filtration systems in washing machines, or subsidies or incentives to adopt filtration or other mitigating technology. Similarly, upgrading or prioritizing wastewater treatment plant filtration could have a more substantial impact on reducing microfiber pollution than individual consumer actions alone. For example, a future project could inform citizen scientists about how water filtration and processing occurs locally,^41^ and then engage them in behaviors that influence policy.

### Limitations

We explicitly tested the generalizability of the sample to urban Dutch residents, but it is unknown how these results would generalize to less urban or non-Dutch consumers. In general, we speculate that citizen scientists would have completed more years of formal education than people who would not volunteer for a time-consuming partnership in scientific studies (as observed here). We speculate that in populations with greater financial and time concerns, it would be more difficult to recruit and retain citizen scientists, but there might also be fewer ceiling effects for variables like environmental concern.

Another key limitation is the small sample size of the citizen scientists, which caused higher variance in the estimates and limited the statistical power to detect small pre-post changes. There was also high drop-out before the follow-up survey, as is typical for effortful citizen science projects in our experience, likely because of the intensiveness of the required tasks about using the fabric bags and collecting and photographing the resulting microfiber sheets. Such dropout is unlikely to have occurred at random, so the dropout could also have affected the pre-post tests. Future work could reduce the participant burden by using a washing machine filter rather than a manual bag or aim to boost retention in other ways like more frequent feedback or higher reimbursement. These issues do not affect the relationships tested in the larger control sample for Hypothesis 3.

Another limitation was the reliance on self-report measures. Behavioral traces would potentially be another way to record how often washing machines are run or with what settings such as load size and temperature. Given the high drop-out rate, the intensity of the procedure, and the challenges in processing the microfiber scans, the citizen science data collection methods could be improved. For example, researchers could use a filtration device attached to the washer that might have advantages in the physical-chemical potential (*e.g.*, capturing fibers from all washed textiles, not just those that were put in the bag). Also, participation across many wash cycles would be a reduced burden for participants, although installing the filtration device could act as a hurdle for participation.

## Conclusions

We assessed the feasibility of citizen scientists monitoring their washing behavior and filtering microplastics at home. Another goal was evaluating the impact of participation on attitudes and future intentions to reduce microplastic emissions (*e.g.*, by using a laundry bag). These goals were overall met. Citizen science appears well-suited to studying household washing behaviors. The main claims of citizen science having transformative potential can be well-tested with impact assessments, validated pre- and post-measures, and focusing on behavior-specific constructs like attitudes towards the effectiveness of the laundry bags. Some of these methods are particularly strong in environmental psychology, so further links may be helpful between researchers studying environmental outcomes and researchers specialized in survey design and behavior measurement. In terms of demography and psychological factors like identity and awareness, citizen scientists are different from the general population, but because those factors were mostly unrelated to washing behaviors in a control group, our study provides initial evidence that such demographic differences may not be a major concern in this domain. It is also possible that measurement or sample issues led to false negative findings, so evidence from other samples and behaviors would be valuable to add more credibility to this claim.

According to a major review, the most effective behavior change interventions targeted behavioral skills, attitudes towards the behavior, and habits.^42^ Our results are broadly consistent with this pattern because factors like environmental awareness and knowledge were largely unchanged by participation as citizen scientists, and largely unrelated to the key washing behaviors that drive environmental impact. This is also consistent with a four-country study showing that psychological factors were mostly unrelated to greenhouse gas emissions from purchasing jeans and t-shirts.^43^ For future research aimed at reducing the environmental impact of microfibers, we suggest less emphasis on changing concerns and knowledge in citizen scientists and the general public. It makes intuitive sense that factors like concern are central to pro-environmental behavior, but in practice, there are increasing claims that environmental behaviors are poorly explained by concern alone.^22^ Instead, we recommend identifying the structural, contextual, and personal factors that might drive behavior, and empirically testing whether they are related and whether citizen science participation affects those behaviors.^18^ Such work has a high potential to identify the most important factors driving washing decisions. Ultimately, reducing household microfiber emissions will be most effective when it combines these insights with top-down environmental policies that reduce fiber emissions and/or improve capture (*e.g.*, at the wastewater treatment level) with less effort by individual consumers.

## Author contributions

Conceptualization (all), data curation (CB, AB, BB, AP, LJ), formal analysis (AB), funding acquisition (CB, AP, LJ), investigation (CB, AB, BB, AP, LJ), methodology (all), project administration (LJ), supervision (CB, AP, LJ), visualization (AB, CB), writing – original draft (AB, CB), writing – review & editing (all). See https://credit.niso.org.

## Conflicts of interest

We report no conflicts of interest.

## Supplementary Material

VA-004-D5VA00037H-s001

## Data Availability

All (anonymized) data, materials, analysis code, and figures are publicly available at https://osf.io/65dz4.
