# Supplementary material for: Psychological outcomes from a citizen science study on microplastics from household clothes washing
Source: Env Sci Adv. 2025 May 13;4(7):1079–93. doi: 10.1039/d5va00037h (PMC12082389; doi:10.1039/d5va00037h)
Supplement: VA-004-D5VA00037H-s001 [file VA-004-D5VA00037H-s001.pdf]

Supplementary Information (SI) for Environmental Science: Advances.
This journal is © The Royal Society of Chemistry 2025

Supplement

Psychological outcomes from a citizen science study on microplastics from household clothes washing

Additional Washing Behaviors

Participants of the control survey (urban Dutch) reported 14 features of their washing behaviors. Only *temperature*, *wears tops*, *wears bottoms*, *days until full load of laundry*, and *washing a full load* were used for the current analyses (see Method). Below are the remaining impacts that were measured and that are not reported in the main text.

Table S1

Additional Washing Behaviors

Behavior	*Measure*
Washing machine	*Top-loading* (1), *front-loading* (2), or *no washing machine* (3)
Garment materials	The material of their three most-worn garments (excluding underwear, socks, towels, and sheets): *natural* (1), *synthetic* (2), *mixed* (3), *other* (4), and *don't know* (5)
Detergent	What type of laundry detergent they mainly use: *liquid detergent* (1), *powdered detergent* (2), *washing pods* (3), *none* (4), or *other* (5)
Softener	How often they use fabric softener: *never* (1) to *always* (5)
Duration wash cycle	How long their most frequently chosen wash cycle takes: *less than an hour* (1), *1-2 hours* (2), *2-3 hours* (3), or *more than three hours* (4)
Spinning speed	Typical spin revolutions per minute: *non-spinning*, *600*, *800*, *900*, *1000*, *1200*, *1400*, or *1600*
Additional program	How often they use an additional program: *never* (1) to *always* (5)
Dryer frequency	How often they use a dryer: *never* (1) to *always* (5)
Filter use	Added an external filter to their washing machine to catch microfibers: *yes* (1), *no* (2), *no (I don't what that is)* (3), or *no (but I added something else to catch microfibers, like a laundry bag)* (4)

Objective knowledge measure

"Synthetic microfibers are one form of microplastics. There are also other types of microplastics, and they are emitted through different sources. Rank the five sources below based on which you think contributes the most to all microplastics in the oceans (1 = most, 4 = least)."

[The options were displayed in random order, and this ordering is correct:]

1. Washing synthetic textiles
2. Tires
3. Plastic packaging
4. Personal care products

We scored the 24 ranking outcomes from low knowledge (1) to high knowledge (5):

1. 4321, 3421, 3412, 4312
2. 3214, 4213, 3124, 4123, 3241, 4231, 4132, 3142
3. 1432, 1423, 1342, 1324, 2314, 2341, 2413, 2431
4. 1243, 2143
5. 1234*, 2134 (* this order is shown above)

Figure S1

Distribution of Video Watching Time in Minutes

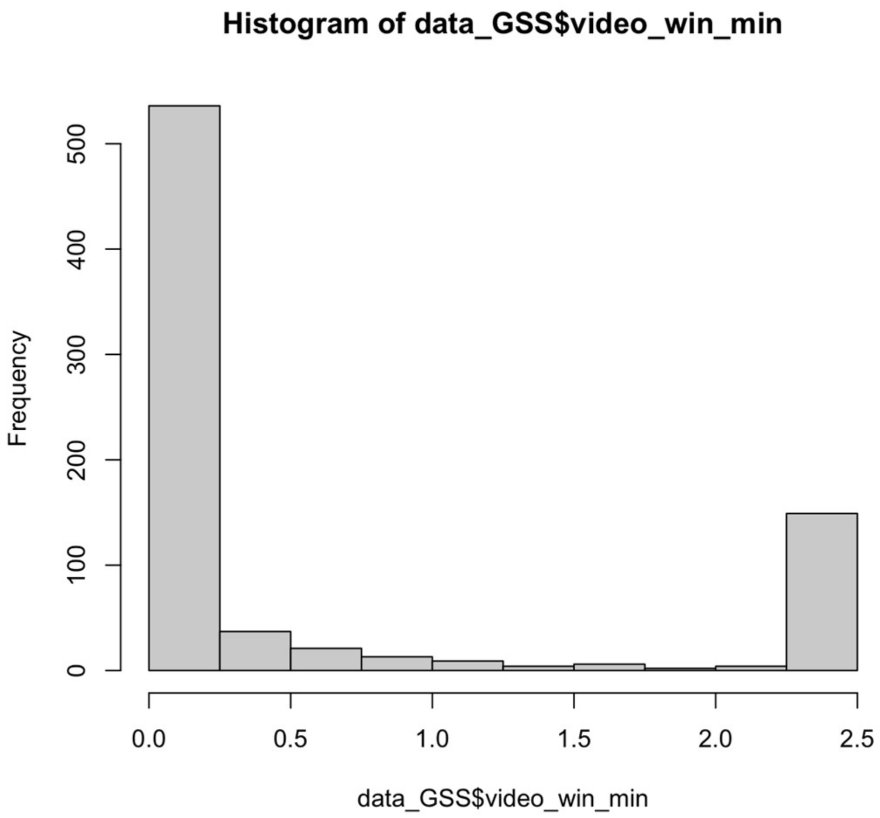

Note. Most participants did not watch the video, as shown by Qualtrics' function 'time spent on page'. 142 participants who stayed on the page longer than the video length were Winsorized to 2.46 minutes.

Figure S2

Distribution of Days Until Laundry

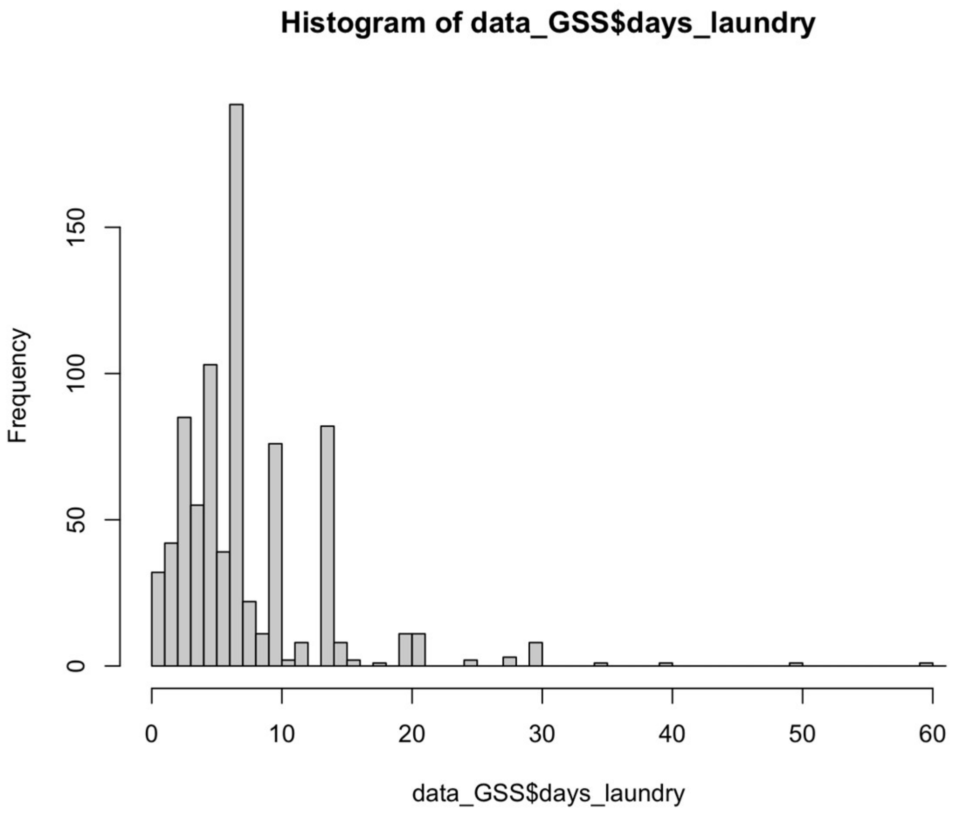

Table S2

Descriptives of Baseline and Post-intervention Scores (Citizen Science Participation, N=56-57)

Variable	Baseline Median (IQR)	Post-intervention Median (IQR)	Baseline *M* (*SD*)	Post-intervention *M* (*SD*)
Social norms	3 (0.5)	3 (1.5)	3.15 (0.6)	3.21 (0.76)
Environmentalist identity	4 (0.75)	4 (0.5)	3.96 (0.65)	4.04 (0.53)
Awareness (harming animals and plants)	4 (1)	5 (1)**	4.16 (1.01)	4.57 (0.68)
Awareness (harming health) robustness check	4 (0.25)	4 (1)**	4.00 (0.74)	4.30 (0.69)
Perceived responsibility	4 (1)	4 (1)**	4.00 (0.74)	4.25 (0.67)
Outcome efficacy (bags reduce harm)	4 (0)	4 (1)**	3.95 (0.59)	3.59 (0.95)
Outcome efficacy (drop in the ocean) robustness check	4 (2)	4 (1)	3.98 (0.94)	4.21 (0.89)
Personal norm (responsibility to act)	4 (1)	4 (1)	3.62 (0.84)	3.61 (0.98)
Personal norm (right thing to do) robustness check	4 (1)	4 (1)	3.71 (0.78)	3.59 (0.76)
Perceived effort	2 (1)	2 (1)	2.36 (0.82)	2.43 (0.95)
Using bags is hard to remember robustness check	2 (1)	2 (1)	2.39 (1.06)	2.57 (1.01)
Bags are affordable robustness check	3 (2)	3 (2)*	3.11 (0.82)	2.77 (0.99)
Bags are durable robustness check	3 (1)	4 (1)***	3.34 (0.55)	3.75 (0.81)
Bags preserve quality exploratory analysis	3 (1)	3 (0)***	3.25 (0.55)	3.18 (0.51)
Bags limit cleaning exploratory analysis	3 (1)	2 (1)	2.75 (0.86)	2.23 (0.66)
Objective microfiber knowledge	2 (1)	2 (1)	2.34 (1.00)	2.38 (1.02)
Intention to use laundry bags	3 (1)	4 (1)	3.5 (0.79)	3.45 (0.99)

Note. IQR = interquartile range. All scores ranged from 1-5, apart from objective and perceived microfiber knowledge (1-4). The results were robust to using alternative analyses apart from bags that are affordable and durable instead of perceived effort, all of which we expected to relate to perceived

behavioral control, and also not robust to outcome efficacy (*drop in the ocean*) instead of outcome efficacy (*reduce harm*).

Table S3

*Descriptives of Baseline Scores of Citizen Science Participants (*N*=56-57) and Control Participants (*N*=715-814)*

Variable	Citizen Scientists Median (IQR)	Control Median (IQR)	Citizen Scientists *M* (*SD*)	Control *M* (*SD*)
Social norms	3 (0.5)	3 (1)	3.15 (0.60)	3.16 (0.79)
Environmentalist identity	4 (0.75)	3.25 (0.75)***	3.96 (0.65)	3.33 (0.73)
Awareness (harming animals and plants)	4 (1)	4 (1)**	4.16 (1.01)	3.90 (0.84)
Awareness (harming health) [robustness check]	4 (0.25)	4 (1)*	4.00 (0.74)	3.74 (0.84)
Perceived responsibility	4 (1)	4 (1)***	4.00 (0.74)	3.61 (0.76)
Outcome efficacy (reduce harm)	4 (0)	4 (1)**	3.95 (0.59)	3.59 (0.87)
Outcome efficacy (drop in the ocean) [robustness check]	4 (2)	4 (1)**	3.98 (0.94)	3.64 (0.95)
Personal norm (responsibility to act)	4 (1)	3 (1)**	3.62 (0.84)	3.25 (0.94)
Personal norm (right thing to do) [robustness check]	4 (1)	4 (1)	3.71 (0.78)	3.50 (0.79)
Perceived effort	2 (1)	3 (2)**	2.36 (0.82)	2.72 (1.02)
Using bags is hard to remember [robustness check]	2 (1)	3 (2)***	2.39 (1.06)	2.90 (0.98)
Bags are affordable [robustness check]	3 (2)	2 (1)***	3.11 (0.82)	2.53 (1.04)
Bags are durable [robustness check]	3 (1)	3 (1)	3.34 (0.55)	3.25 (0.70)
Bags preserve quality	3 (1)	3 (1)	3.25 (0.55)	3.22 (0.73)
Bags limit cleaning	3 (1)	3 (1)**	2.75 (0.86)	3.07 (0.87)
Objective microfiber knowledge	3 (1)	2 (1)	2.46 (0.81)	1.85 (0.75)
Intention to use laundry bags [exploratory]	2 (1)	2 (2)***	2.34 (1.00)	2.15 (0.94)

Note. IQR = interquartile range. All scores ranged from 1-5, apart from objective and perceived microfiber knowledge (1-4). The results were robust to using alternative items apart from personal norm (*right thing to do*) instead of personal norm (*responsibility to act*) and bags are durable instead of perceived effort, both of which we expected to relate to perceived behavioral control.

Figure S3

Citizen Science Effects (Robustness Checks)

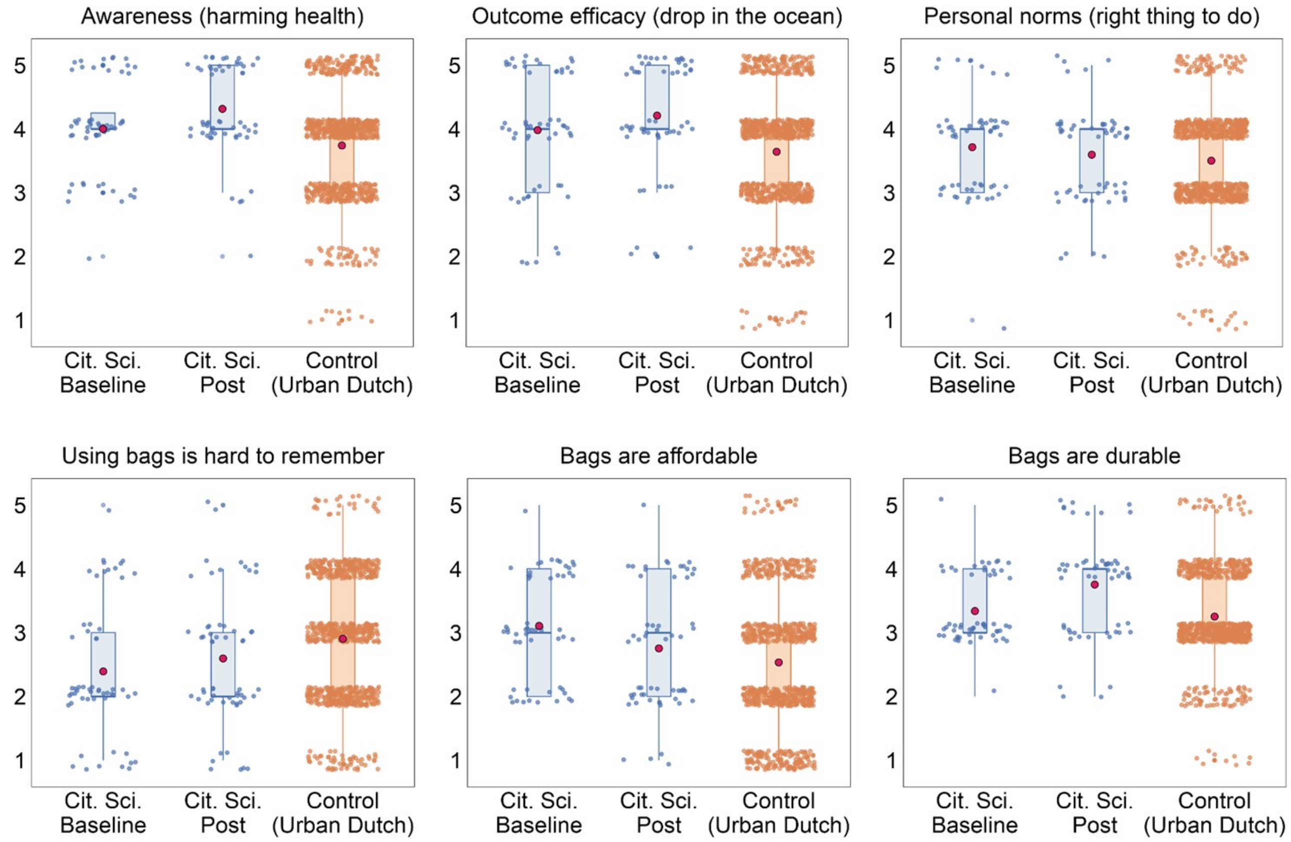

Note. The red dots indicate means, the boxes indicate interquartile ranges, and the lines in the boxes indicate medians. The points are jittered and partially transparent to better show the distributions.

Figure S4

Citizen Science Effects (Exploratory Analyses)

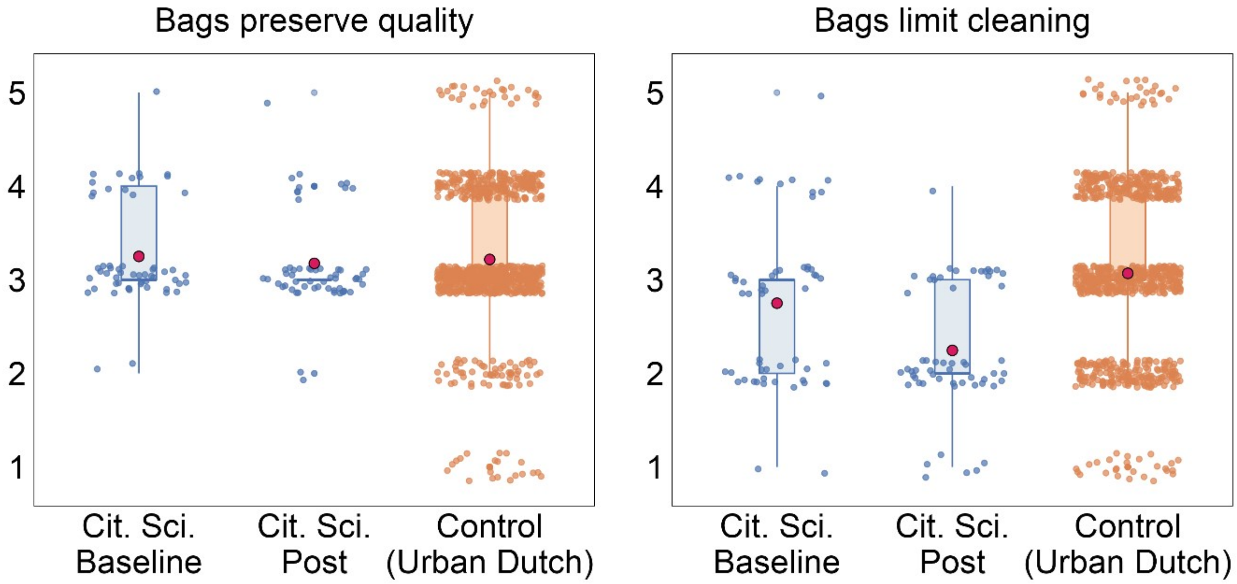

Note. The red dots indicate means, the boxes indicate interquartile ranges, and the lines in the boxes indicate medians. The points are jittered and partially transparent to better show the distributions.

Table S4

*Spearman Correlations of the Intention to Use Laundry Bags, Video-Watching, and Washing Behaviors in the Control Survey (*N*s = 768-814)*

	Intention to use laundry bags	Video-watching (sec)	Temperature (°C)	Wears tops (freq.)	Wears bottoms (freq.)	Washing a full load (¼ to full)	Days until full load
	2.92 (0.97)	0.63 (0.93)	40.99 (9.39)	2.03 (0.68)	2.5 (0.69)	0.84 (0.18)	7.72 (5.78)
	1-5	0.02-2.46	20-90.5	1-3	1-3	0-1	1-60
Video-watching	.04						
Temperature	.06	.01					
Wears tops	-.07	.11	-.05				
Wears bottoms	-.10	.11	-.14	.37			
Washing a full load	-.09	.06	.00	.04	.10		
Days until full load	-.05	.12	-.11	.26	.31	.16	

Note. Spearman's rho correlations (r_s). Correlations $|r_s| \geq .08$ are significant at $p < .05$, $|r_s| \geq .10$ at $p < .01$, and $|r_s| \geq .13$ at $p < .001$.

Table S5

*Robustness Checks (without Exclusions): Spearman Correlations between Psychological Variables and the Intention to Use Laundry Bags and Video-watching in the Control Survey (*N*s = 688-821)*

			Intention to use laundry bags	Video-watching (sec)
		Range	1-5	0.02-2.46
		M (SD)	2.93 (0.98)	.62 (0.93)
	Range	*M (SD)*		
Social norms	1-5	3.17 (0.79)	.31	.07
Environmentalist identity	1-5	3.34 (0.73)	.36	.17
Awareness (harming animals and plants)	1-5	3.90 (0.85)	.21	.14
Awareness (harming health) robustness	1-5	3.74 (0.84)	.26	.08
Perceived responsibility	1-5	3.62 (0.76)	.31	.09
Outcome efficacy (reduce harm)	1-5	3.59 (0.87)	.41	.09
Outcome efficacy (drop in the ocean) robustness	1-5	3.64 (0.95)	-.13	.01
Personal norm (responsibility to act)	1-5	3.26 (0.94)	.57	.12
Personal norm (right thing to do) robustness	1-5	3.51 (0.80)	.55	.07
Perceived effort	1-5	2.73 (1.03)	-.19	-.13
Using bags is hard to remember robustness	1-5	2.91 (0.99)	-.20	-.14
Bags are affordable robustness	1-5	2.55 (1.05)	.43	.01
Bags are durable robustness	1-5	3.26 (0.71)	.22	.02
Bags preserve quality	1-5	3.22 (0.73)	.32	.04
Bags limit cleaning	1-5	3.08 (0.87)	-.22	-.05
Perceived microfiber knowledge	1-4	1.85 (0.75)	.16	.08
Objective microfiber knowledge	1-4	2.16 (0.94)	.09	.00

Note. Spearman's rho correlations (r_s). Correlations $|r_s| \geq .07$ are significant at $p < .05$, $|r_s| \geq .10$ at $p < .01$, and $|r_s| \geq .13$ at $p < .001$.

Table S6

*Robustness Checks (without Exclusions): Correlations between Psychological Variables and Washing Behaviors in the Control Survey (Urban Dutch) (*N*s = 710-834)*

			Temperature (°C)	Wears tops (freq.)	Wears bottoms (freq.)	Washing a full load (¼ to full)	Days until full load
	Range		20-95	1-3	1-3	1-4	1-60
		M (*SD*)	41.7 (10.3)	2.03 (0.68)	2.50 (0.69)	0.84 (0.18)	7.66 (5.74)
Social norms	1-5	3.17 (0.79)	.02	-.01	.00	.01	-.04
Environmentalist identity	1-5	3.34 (0.73)	-.01	.06	.08	.07	.09
Awareness (harming animals and plants)	1-5	3.90 (0.85)	-.02	.07	.17	.16	.17
Awareness (harming health) robustness	1-5	3.74 (0.84)	-.02	.06	.10	.08	.08
Perceived responsibility	1-5	3.62 (0.76)	-.04	.07	.15	.12	.11
Perceived microfiber knowledge	1-4	1.85 (0.75)	.01	.07	.09	.06	.04
Objective microfiber knowledge	1-5	2.16 (0.94)	-.03	-.02	-.05	.03	-.05

Note. Spearman's rho correlations (r_s). Correlations $|r_s| \geq .07$ are significant at $p < .05$ (apart from the correlation between environmentalist identity and washing a full load), $|r_s| \geq .10$ at $p < .01$, and $|r_s| \geq .13$ at $p < .001$.

Table S7

*Robustness checks: Spearman Correlations between Demographics and the Intention to Use Laundry Bags and Video-watching and Washing Behaviors in the Control Survey (Hypothesis 3) (*N*s = 687-1052)*

			Gender	Age	Education
	Range		0-1	16-88	1-6
		M (SD)	0.61 (0.49)	48.6 (16.6)	2.90 (1.46)
Intention to use laundry bags	1-5	2.93 (0.98)	.12	-.09	.09
Video-watching (sec)	0.02-2.46	0.62 (0.93)	-.01	.43	-.09
Temperature (°C)	20-95	41.7 (10.3)	.01	-.01	-.02
Wears tops (freq.)	1-3	2.03 (0.68)	-.07	.26	.01
Wears bottoms (freq.)	1-3	2.50 (0.69)	-.02	.12	.13
Washing a full load (¼ to full)	1-4	0.84 (0.18)	.06	.02	.11
Days until full load	1-60	7.66 (5.74)	-.08	.11	.14

Note. Spearman's rho correlations (r_s). Correlations $|r_s| \geq .07$ are significant at $p < .05$, $|r_s| \geq .10$ at $p < .01$, and $|r_s| \geq .13$ at $p < .001$.

Retention and Attrition (Citizen Science Sample)

Number of participants who:	
99	Registered
95	Received (by picking up or via post) the package with supplies to participate
62	Started the study
57	Did 3 or more washes
45	Did 5 or more washes
34	Did 8 or more washes
24	Did 10 or more washes
11	Did 12 washes

Note. A wash was counted when both the pre- and post-wash surveys were filled out and uploaded. Many washes (72) were pre-surveyed but not post-surveyed. A total of 62 participants sent back their fabric sheets by post. The analyzed sample of 57 was derived from the participants who completed at least three pre- and post-wash surveys.
